# Hemolytic Anemia and Reactive Thrombocytosis Associated With Cefoperazone/Sulbactam

**DOI:** 10.3389/fphar.2019.01342

**Published:** 2019-11-08

**Authors:** Ling Zhou, Jianan Bao, Jingjing Ma

**Affiliations:** Department of Pharmacy, The First Affiliated Hospital of Soochow University, Soochow University, Suzhou, China

**Keywords:** adverse reaction, cefoperazone/sulbactam, immune hemolytic anemia, reactive thrombocytosis, probable

## Abstract

**Background:** Cefoperazone/sulbactam is a broad-spectrum antibacterial agent. Drug-induced immune hemolytic anemia is a rare but serious condition, and reactive thrombocytosis is caused by processes extrinsic to the megakaryocyte. Limited data are available for cefoperazone/sulbactam-associated hemolytic anemia and reactive thrombocytosis.

**Case presentation:** We report the case of a 60-year-old woman undergoing surgical excision of the left atrial myxoma, who presented with hemolytic anemia and thrombocytosis following cefoperazone/sulbactam administration for lung infection. The duration of cefoperazone/sulbactam therapy was 8 days. Blood analysis showed markedly decreased hemoglobin, hematocrit, and red blood cell levels, with elevated lactate dehydrogenase, indirect bilirubin, platelets, and reticulocytes. Furthermore, the direct antiglobulin test was positive for anti-C3 and a diagnosis of hemolytic anemia and reactive thrombocytosis was made. Then, cefoperazone/sulbactam was discontinued and red blood cell transfusion was performed for 3 days. After 1 week, the patient’s condition improved, and she was discharged.

**Conclusion:** This is the first suspected case report of immune hemolytic anemia and reactive thrombocytosis related to cefoperazone/sulbactam. Caution should be taken for this reaction in patients undergoing cefoperazone/sulbactam therapy.

## Introduction

Patients with autoimmune hemolytic anemia may present with symptoms of anemia, hemolysis or symptoms of an underlying disorder. (Hill et al., 2017) Drug-induced immune hemolytic anemia (DIIHA) is a rare but potentially life-threatening condition that occurs in approximately one in a million cases. The drugs implicated include antibiotics (e.g., piperacillin, cefotetan, ceftriaxone), antineoplastics (e.g., fludarabine, oxaliplatin), and NSAIDs (e.g., acetaminophen, diclofenac). (Garratty and Arndt, 2014)

Cefoperazone/sulbactam, a third-generation cephalosporin, is a broad-spectrum β-lactam/β-lactamase inhibitor combination with a number of clinical uses including upper and lower respiratory infection, urinary tract infection, and peritonitis. (Gao et al., 2016; Su et al., 2018)

The patient reported here was admitted for myxoma surgical excision and was prescribed piperacillin/tazobactam for lung infection at admission. After the lung infection worsened, the patient was shifted to cefoperazone/sulbactam. Herein, we describe the first suspected case of hemolytic anemia and reactive thrombocytosis in response to cefoperazone/sulbactam in a patient with lung infection.

### Case Presentation

The patient was a 60-year-old woman who presented to cardiovascular surgery for left atrial myxoma excision. At admission, the patient was diagnosed with lung infection by chest radiography performed outside the hospital, accompanied with productive cough. Blood test showed the white blood cell count (WBC) 17.22 × 10^9^/L (normal 3.5–9.5 × 10^9^/L), neutrophil count (NE) 14.29 × 10^9^/L (normal 1.8–6.3 × 10^9^/L), and C-reactive protein (CRP) 10.63 mg/L (normal 0–3 mg/L). Vital signs were normal. Therefore, piperacillin/tazobactam was prescribed. She had no history of hematological disorders and laboratory testing showed hemoglobin, platelet, lactate dehydrogenase (LDH), and indirect bilirubin (IBIL) levels within normal ranges. Preoperative examinations were performed and indicated no surgical contraindications. The patient underwent successful complete excision of left atrial myxoma.

**Figure 1:**
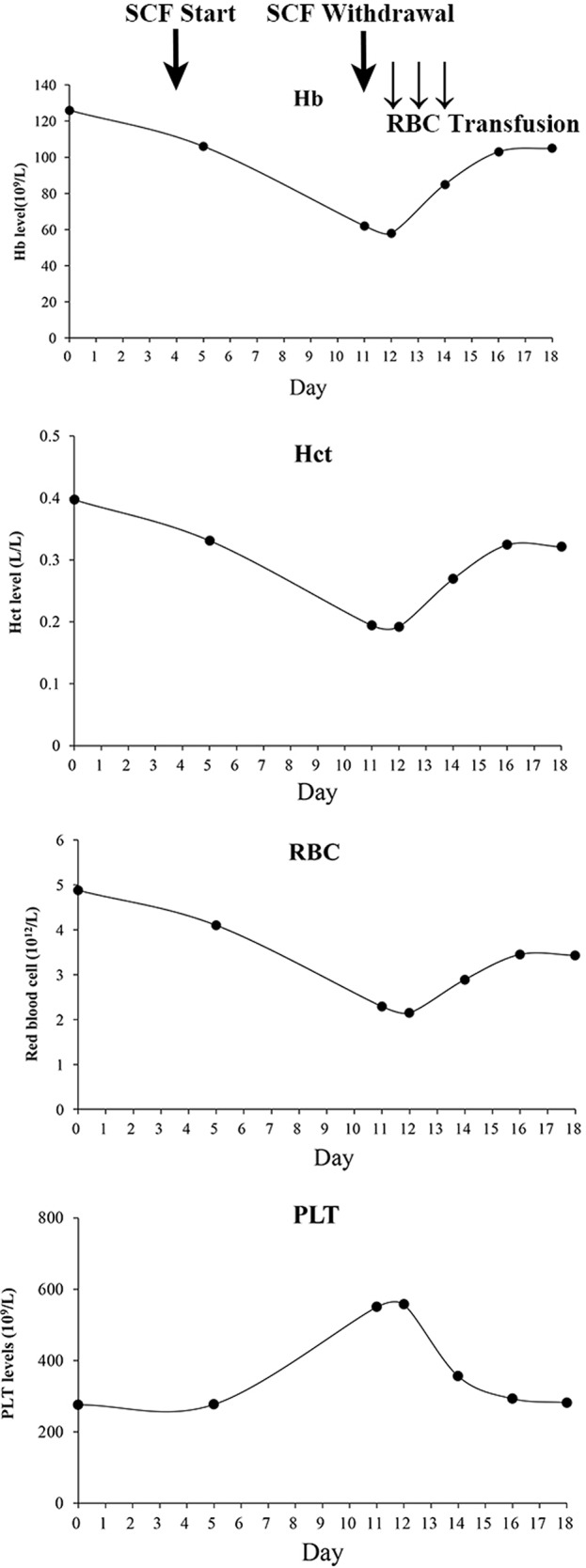
Serial changes of complete blood count during cefoperazone/sulbactam therapy and withdrawal. SCF, Cefoperazone/sulbactam; Hb, hemoglobin; RBC, Red blood cell; Hct, Hematocrit; PLT, Platelet.

Ten days later, her lung infection deteriorated with her temperature at 38.5̊ C, and computed tomography imaging showed pneumonia, warranting the switching of therapy to cefoperazone/sulbactam (3 g, q12h). On the second day of cefoperazone/sulbactam therapy, laboratory analysis showed a slight reduction in hemoglobin (115 g/L, normal 130–175 g/L) and hematocrit (0.331 L/L, normal 0.4–0.5 L/L). Platelet and red blood cell (RBC) counts were 277 × 10^9^/L (normal 125–350 × 10^9^/L) and 4.1 × 10^12^/L (4.3–5.8 × 10^12^/L), respectively. On the third day, the blood culture was obtained with no bacterial growth. On the 7th day, hematocrit, hemoglobin, and RBC decreased to 0.194 L/L, 62 g/L, and 2.29 × 10^12^/L respectively, whereas the platelets were elevated to 550 × 10^9^/L. Blood assessment revealed that LDH was 356.8 U/L (normal, 120–250 U/L) and IBIL was 27 µmol/L (normal, 1.7–10.2 µmol/L). The situation was worse on the following day: hematocrit, hemoglobin, and RBC fell to 0.192 L/L, 58 g/L, and 2.15 × 10^12^/L, respectively, with platelet rising to 557 × 10^9^/L. Meanwhile, the vital signs were all normal. Then, cefoperazone/sulbactam was ceased and substituted with ticarcillin/clavulanate (3.2 g, q8h); furthermore, daily transfusion of packed RBC (2 units) was commenced for 3 days. The levels of hematocrit, hemoglobin, and RBCs then elevated to 0.269 L/L, 85 g/L, and 2.89 × 10^12^/L, respectively, and the platelets decreased to 356 × 10^9^/L (**Figure 1**). RBC transfusion was discontinued, and the hematologist reviewed the patient with suspected DIIHA. The blood samples were then sent for serologic tests. A warm direct antiglobulin test (DAT) returned positive for anti-C3 (1:64) and negative for anti-IgG, G6PD/6PGD was 1.05 (normal, 1–2.3), reticulocytes were 8.1% (normal, 0.5%–1.5%). Based on the clinical, laboratory, and serologic findings, a diagnosis of DIIHA and thrombocytosis was made.

No further interventions were made to manage hemolytic anemia and thrombocytosis. After five days, laboratory analysis showed gradual remission of hematocrit (0.336 L/L), hemoglobin (108 g/L), RBC (3.56 × 10^12^/L), IBIL (5.9 µmol/L), LDH (175.3 U/L), and platelets (276 × 10^9^/L). Lung infection was effectively controlled with WBCs at 8.3 × 10^9^/L, NE at 5.81 × 10^9^/L, and temperature at 36.1̊ C without cough; ticarcillin/clavulanate was then discontinued. Within 2 more days, favorable results were presented with hematocrit (0.345 L/L), hemoglobin (111 g/L), RBC (3.61 × 10^12^/L), reticulocytes (2.9%), and platelets (263×10^9^/L). One day later, the patient was discharged with stable post-operative function and normal vital signs.

One week later, the patient’s levels of hematocrit, hemoglobin, RBCs, and reticulocytes were all within normal ranges. The patient’s recovery was uneventful.

## Discussion

Cefoperazone/sulbactam was the most possible factor that induced hemolytic anemia and reactive thrombocytosis in this case. A PubMed search of “cefoperazone/sulbactam AND hemolytic anemia” yielded only one previous report of immune hemolytic anemia associated with cefoperazone/sulbactam. (Baek et al., 2009) In that case, immune hemolytic anemia was first caused by ceftizoxime, and substitution with cefoperazone/sulbactam further worsened the condition, with no platelet involvement. This report represents the first case of independent cefoperazone/sulbactam-induced hemolytic anemia and unique reactive thrombocytosis.

Hemolytic anemia is characterized as anemia due to shortened survival of circulating RBCs. The key clues that suggest hemolytic anemia include low hemoglobin levels diagnosed as anemia, an increase in reticulocyte count and positive RBC antibodies. Patients may also show increased LDH and IBIL and decreased haptoglobin levels. (Chinese Society of Hematology, Chinese Medical Association, 2017)

DIIHA results from immunization against the drug and/or RBCs and is identified by clinical evidence of hemolysis associated with drug therapy. Drugs are small molecular weight chemicals that could become immunogenic if complexed with a carrier molecule. Drugs are haptens that need carriers such as proteins, RBCs, or the platelet membrane to evoke an antibody response. (Shulman and Reid, 1993; Garratty, 2012) A drug etiology should be considered for newly diagnosed IHA and a careful history of medication must be taken. (Garbe et al., 2011) Over 130 drugs have been implicated in DIIHA and an indisputable evidence would require: a well-defined hemolytic anemia, temporal relationship to drug therapy, positive DAT after drug therapy, and improved hematological response in the patient after drug cessation. (Gao et al., 2016) The patient here met the above criteria used to determine DIIHA evaluation.

Meanwhile, the patient showed concurrent thrombocytosis during cefoperazone/sulbactam therapy. Thrombocytosis showed a temporal relationship with cefoperazone/sulbactam administration and when cefoperazone/sulbactam stopped, the platelet levels were restored. Reactive thrombocytosis, also described as secondary thrombocytosis, accounts for most cases of thrombocytosis, and hemolytic anemia is one of the common causes. (Tefferi, 2018) A pediatric patient with AIHA was reported to show thrombocytosis. (Jea et al., 2007) Kobayashi et al. reported that ribavirin treatment in chronic hepatitis C patients induced anemia and may have led to increases in endogenous serum erythropoietin that in turn resulted in the stimulation of platelet production. (Kobayashi et al., 2012) During cefoperazone/sulbactam therapy and withdrawal, the patient’s platelets showed an opposite change tendency compared to hemoglobin, RBCs, and hematocrit (**Figure 1**), suggestive of reactive thrombocytosis due to hemolytic anemia.

The limitation of the case was a lack of serological evidence by which we could differentiate the mechanism of DIIHA, for example nonimmunologic protein adsorption or immune-complex. (Arndt, 2014) Second, the levels of hematocrit, hemoglobin, and RBCs were elevated after packed RBC transfusion, and not merely due to cefoperazone/sulbactam withdrawal. From the clinical point of view, treatment with cefoperazone/sulbactam was the only probable cause of the hemolytic anemia. The probability of adverse drug reaction was assessed using the Naranjo algorithm. (Naranjo et al., 1981) A score of +6 was predictive of cefoperazone/sulbactam being the culprit.

## Conclusion

This is the first suspected case associating immune hemolytic anemia and reactive thrombocytosis with cefoperazone/sulbactam. Using the Naranjo scale, cefoperazone/sulbactam was considered the probable cause of the episode. Close monitoring of blood assessment over the course of cefoperazone/sulbactam treatment is thus highly recommended.

## Data Availability Statement

The raw data supporting the conclusions of this manuscript will be made available by the authors, without undue reservation, to any qualified researcher.

## Ethics Statement

The study was approved by the ethical committee of the First Affiliated Hospital of Soochow University. Consent for publication was obtained from the patient.

## Author Contributions

LZ collected data. JM performed data analysis and wrote the first draft of the manuscript. JB reviewed the manuscript and made critical revisions. All authors read and approved the final version of the manuscript.

## Conflict of Interest

The authors declare that the research was conducted in the absence of any commercial or financial relationships that could be construed as a potential conflict of interest.

## Abbreviations

DIIHA, drug-induced immune hemolytic anemia; LDH, loactate dehydrogenase; IBIL, indirect bilirubin; RBC, red blood cell; DAT, direct antiglobulin test.
